# Utilization of Prognostic Biomarker Soluble Urokinase Plasminogen Activator Receptor in the Emergency Department: A Tool for Safe and More Efficient Decision-making

**DOI:** 10.1177/11772719221081789

**Published:** 2022-03-09

**Authors:** Ria M Holstein, Marja T Mäkinen, Maaret K Castrén, Johanna M Kaartinen

**Affiliations:** Department of Emergency Medicine and Services, Helsinki University Hospital and University of Helsinki, Helsinki, Finland

**Keywords:** Soluble urokinase plasminogen activator receptor (suPAR), discharges, admission, triage, risk stratification, emergency department, biomarkers, prognostic biomarkers, aged

## Abstract

**Introduction:**

Risk stratification in the emergency departments (EDs) is in critical need for new applications due to ED overcrowding and hospitalization of older people. We aimed to evaluate the expediency, efficiency and safety of a prognostic biomarker, soluble urokinase plasminogen activator receptor (suPAR), as a tool for the risk assessment of patients arriving at the ED.

**Methods:**

We performed a comparative cross-sectional study in 2 emergency departments (EDs), suPAR measurements being incorporated into routine blood sampling in the intervention ED. The primary outcome was the number of discharges from the ED. The importance of the outcomes was examined by appropriate multi- or bivariate analysis.

**Results:**

The absolute and relative number of discharges were similar between the intervention and control groups [121 (55.3%) vs 62 (55.9%)]. No significant differences between the groups were seen in the length of stays in the ED. Patients with low suPAR values were more likely discharged and patients with high suPAR values more likely admitted to hospital. Two admitted patients with low suPAR values could have been discharged safely.

**Conclusion:**

The utilization of suPAR did not increase the risk for neither positive nor negative outcomes. Low suPAR values could be potential in discharging more patients safely. Instead of unselected patient populations, the benefits of suPAR measurements in the ED could emerge in the assessment of a more precisely determined and selected group of patients.

## Introduction

Emergency department (ED) overcrowding is a significant issue discussed all over the world, resulting in increased patient mortality rates, decreased treatment quality, impaired hospital access, prolonged lengths of stays as well as financial loss. The future perspective of the overcrowded EDs and hospital beds is concerning due to population growth, aging population and consequential increases in the volume of patients seeking care from the EDs.^1-3^

In addition to clinical examination, the risk stratification in the EDs relies principally on various vital sign-dependent scores, “track-and-trigger-scores,” such as New Early Warning Score (NEWS). Nevertheless, these scores fail to identify patients with normal vital signs but with high risk of severe illness: a former study suggests that equal triage levels are not necessarily indicators of equal odds of death.^4,5^ Therefore, more supporting tools for identifying and classifying patients in the EDs are needed.^6^ By allowing more safe discharges in a shorter period of time, the patient flow could be improved and the overcrowding avoided, thus not only providing the ED with increased amount of hospital beds and resources but also making it a safer place for all patients.^7^

For the issue, prognostic biomarkers may be a prospective solution. They are suggested to be potential tools for more efficient patient assessment and risk stratification in the ED when incorporated into other clinical information.^8-10^ *Soluble urokinase plasminogen activator receptor* (suPAR) has shown to be a strong indicator of the presence, severity and future development of numerous acute diseases.^6,7,11-13^ SuPAR is a soluble form of a membrane-bound receptor, urokinase plasminogen activator receptor (uPAR), which is found on the surface of various immunoactive cells. SuPAR is formed when uPAR is cleaved from the cell’s plasma membrane and released into body solvents such as plasma, serum, and urine (from which suPAR can be localized and measured).^2,14^ The suPAR value of healthy individuals is cited to be <4 ng/mL. The suPAR value of over 6 ng/mL at the time of index admission indicates a remarkable higher risk of negative outcomes when used independently and with NEWS scoring.^6,15^ Furthermore, regardless of not being considered as an acute phase reactant, a high suPAR value in the acute clinical setting is suggested to be associated with higher long-term readmission rates and increased risk of mortality.^16^ Low suPAR values are, in turn, associated with lower readmission rates and lower risks of mortality.^16,17^

Previous literature suggests that suPAR values increase with age and certain comorbidities such as diabetes mellitus, cardiovascular diseases, chronic obstructive pulmonary disease (COPD), and chronic kidney disease (CKD). However, the risk-predicting value of suPAR is considered not to be affected by neither the age-related differences nor the existence of these comorbidities.^18-22^ When used both independently and with ASA classification, suPAR can also predict postoperative complications and mortality in surgical patients regardless of the underlying comorbidities.^23^

Our study aims to evaluate the expediency and safety of suPAR as a tool in the ED’s risk stratification and patient assessment. We hypothesize that in the decision-making of an unselected patient population arriving at the ED, suPAR values would act as a supporting tool in the process of discharging or admitting the patient. Simultaneously, our study hypothesizes that suPAR values would improve patient safety by identifying the patients requiring special attention, resulting in increased amount of discharges, diminished “safe side” admissions, similar or shorter index admission times and through lessened ED crowding, to significant cost reductions.

## Materials and Methods

### Study design and participants

The study was a comparative cross-sectional study operated in the Emergency Medicine and Services Unit of Helsinki University Hospital. The research was performed between 11 May 2020 and 24 May 2020 in 2 separate emergency departments, one serving as the intervention group and one as the control.

As our data, we used an unselected population of patients from the intervention and control departments. The inclusion criteria for the patients were admission to the ED, written consent and a chief complaint requiring a venous blood sample. A patient was excluded if the ED visit did not involve blood sampling, the blood sample for the suPAR analysis wasn’t received or if the patient did not consent to the study.

The study’s covariates included sex, age, vital signs, infection variates (necessity of antibiotics, the antibiotic, infection and the causing microbe), routine blood counts (sodium, creatinine, leukocytes, plasma glucose, hemoglobin), C-reactive protein (CRP), D-dimer, lactate, and SARS-CoV-2 PCR result.

All information was sought from electronic patient record systems of Helsinki University Hospital.

### Algorithm

**Figure 1.**
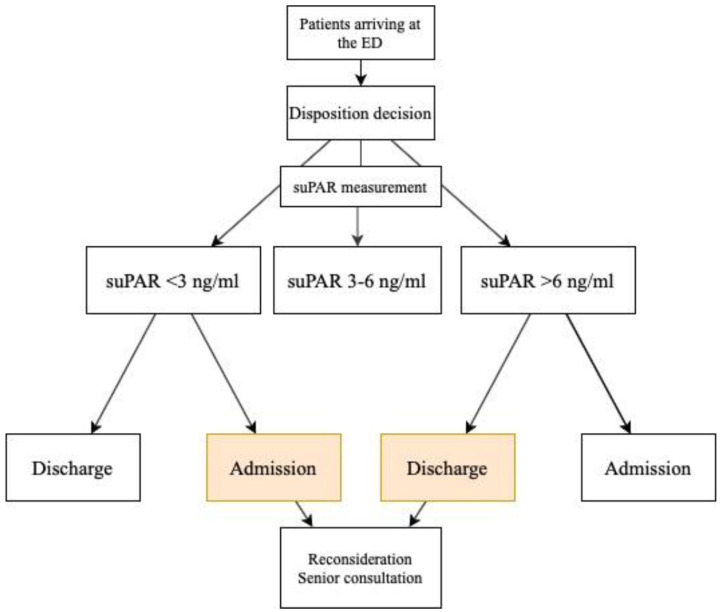
Proposed algorithm of the implementation of suPAR into routine blood sampling.

The staff of the intervention ED were introduced to the algorithm’s 3 main principles: (1) low suPAR level is associated with a lower risk of life-threatening disease and supports the decision of discharge, (2) elevated suPAR levels are associated with a higher risk of life-threatening disease, and (3) when admitting the patient with a particularly low suPAR value (<3 ng/mL) or discharging a patient with a high value (>6 ng/mL), a reconsideration should be made involving a senior consultant. The staff of the control ED were not familiarized with the algorithm (Figure 1).

### Intervention

The intervention being suPAR, the suPAR measurements were incorporated into routine blood sampling. Plasma samples for the analysis were collected by the laboratory personnel between 8 a.m. and 7:30 p.m. The actual measurement was carried out with quantitative suPARnostic^®^ Quick Triage-point-of-care-tests and the research assistants were familiarized with the analyzation process via online orientation by the manufacturers (Virogates A/S, Denmark). The actual test results were presented to the physician by a written document when the preliminary care decisions were made.

In the control group, blood samples were collected but suPAR values were blinded from the ED physicians.

### Outcomes

The primary outcome researched was the number of discharges from the ED within 24 hours. The secondary outcomes were (1) length of stay in the ED, (2) number of hospital admissions, (3) length of stay during hospital admission, (4) number of readmissions to hospital, and (5) all-cause mortality. The follow-up time was originally determined as 30 days but later changed to 7 days as it was in line with our study’s aspects and thus gave more realistic results. Readmissions were identified as the discharged patients’ non-elective admissions to the ED during the follow-up.^12^

### Statistical analysis

For the analysis of the data, the significance test was used. Results are presented as numbers (n) and percentages (%) for categorial variables and as mean and as median and interquartile range (25th-75th percentile) for continuous variables. A *P*-value less than .05 was considered statistically significant. Comparisons between the study groups were performed using Mann-Whitney-*U*-test for continuous variables and Fisher’s test for categorial variables. The power calculation was based on chi-squared test by simulating the final data due to significant changes in the sample size (See Limitations Section, p. 10). All statistical analyses were performed with SPSS software 25.0.

### Ethics approval

The study was approved by the Ethical Committee of the Helsinki University Hospital (Ref. no. § 33 HUS/141/2020 and § 32 HUS/3346/2019) The committee decided that the study could be carried out if a consent was received from the patient. The patients were informed of the nature of the study.

## Results

The median age of the whole study population was 67 years (25-75th percentile 52-78) and 192/330 (58.1%) were women. The intervention group consisted of 219 patients with a median age of 67 years, 58.4% being women. The control group consisted of 111 patients with a median age of 68 years, 57.6% being women (Table 1). The proportion of patients discharged within 24 hours of index admission was 55.3% (121/219) in the intervention group and 55.9% (62/111) in the control group (*P* = 1.00) (Table 2).

**Table 1.** Characteristics of the patient populations in intervention and control groups.

	All	Intervention ED	Control ED	*P*-value
N (%)/median (25-75th percentile)	330 (100)	219 (66.4)	111 (33.6)	
Female sex	192 (58.1)	128 (58.4)	64 (57.6)	.91
Age, y	67 (52-78)	67 (51-78.5)	68 (53-77)	.90
suPAR, ng/mL	3.9 (2.9-5.68)	3.6 (2.85-5.4)	4.5 (3-6.25)	.06
CRP, mg/L	4 (4-23)	4 (4-20)	4 (4-24.5)	.93
Lactate, g/mL	1.1 (0.9-1.8)	1 (0.9-1.5)	1.8 (1.75-1.93)	.06
Creatinine, mL/min	72 (58-89)	73 (60-91)	64.5 (57-84.75)	.09
Leukocytes, xE9/L	7.7 (6-10.22)	7.65 (5.9-10.5)	7.7 (6.03-9.55)	.98
Hemoglobin, g/L	131 (118-143)	132 (119-144.25)	129.5 (113.25-140.75)	.18
Sodium, mmol/L	141 (138-143)	141 (138-143)	141 (138-143)	.82
NEWS score	0 (0-2)	0 (0-2)	0 (0-1)	.01
Preliminary diagnosis, N (%)				
Infectious	13 (3.9)	7 (3.2)	6 (5.4)	.37
Cancer and neoplasms	9 (2.7)	6 (2.7)	3 (2.7)	1.00
Endocrinologic and metabolic	2 (0.6)	2 (0.9)	0 (0.0)	.55
Neurological	7 (2.1)	5 (2.3)	2 (1.8)	1.00
Ophthalmological	1 (0.3)	1 (0.5)	0 (0.0)	1.00
Cardiovascular	63 (19.1)	44 (20.1)	19 (17.1)	.56
Respiratory	14 (4.2)	10 (4.6)	4 (3.6)	.78
Gastrointestinal	32 (9.7)	28 (12.8)	4 (3.6)	.01
Dermatological	3 (0.9)	1 (0.5)	2 (1.8)	.26
Musculoskeletal	17 (5.2)	11 (5.0)	6 (5.4)	1.00
Urogenital	17 (5.2)	9 (4.1)	8 (7.2)	.29
Not classified symptoms	125 (37.9)	78 (35.6)	47 (42.3)	.28
Injuries and poisoning	11 (3.3)	10 (4.6)	1 (0.9)	.11
Specific cases	6 (1.8)	1 (0.5)	5 (4.5)	.02

Abbreviations: CRP, C-reactive protein; NEWS, National Early Warning Score; suPAR, soluble urokinase plasminogen activator receptor.

Data are presented as numbers and percentages (%) and medians (25-75th percentile), as appropriate.

**Table 2.** Outcomes of the patient populations in intervention and control groups.

N (%)/median (25-75th percentile)	All	Intervention ED	Control ED	*P*-value
	330 (100)	219 (66.4)	111 (33.6)	
Discharges within 24 h	183 (55.5)	121 (55.3)	62 (55.9)	1.00
Admissions to hospital	147 (44.5)	98 (44.7)	49 (44.1)	1.00
Readmissions at 7 d	12/183 (6.6)	9 (4.1)	3 (2.7)	.76
Length of stay in the ED, h	4.6 (3.4-6.2)	4.5 (3.5-6.1)	4.9 (3.2-6.4)	.82
Length of stay in the hospital, d	0 (0-2)	0 (0-1)	0 (0-3)	.17
suPAR, patients alive at 7 d, ng/mL	3.9 (3.4-6.2)	3.6 (2.8-5.4)	4.5 (3.0-6.3)	.06
suPAR, patients dead at 7 d, ng/mL	4.8	4.8	No patients	

Abbreviation: suPAR, soluble urokinase plasminogen activator receptor.

Data are presented as numbers and percentages (%) and medians (25th-75th percentile), as appropriate.

**Figure 2.**
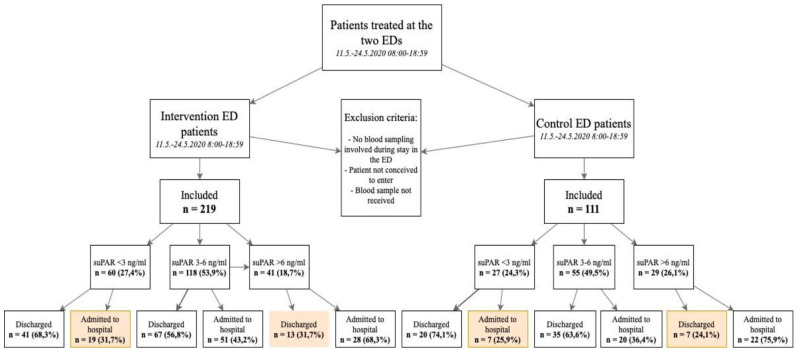
Flow diagram of the study’s patient population.

To evaluate the expediency of suPAR values in the ED’s decision making, an algorithm was implemented in the intervention ED. The algorithm divided included patients in 3 groups according to the measured suPAR values: (1) suPAR under 3 ng/mL, (2) suPAR between 3 and 6 ng/mL, and (3) suPAR over 6 ng/mL. These patients were further investigated and divided into 2 subgroups with a contradiction between the suPAR value and the decision of follow-up treatment. (Marked as orange in the figures). Of all patients with suPAR less than 3 ng/mL, 70.1% (61/87) were discharged directly from the ED. On the contrary, of all patients with suPAR values greater than 6 ng/mL, only 28.6% (20/70) were directly discharged. From another point of view, of those with suPAR values greater than 6 ng/mL, 71.4% (50/70) were admitted to hospital, while the proportion was 29.9% (26/87) in the patient group with suPAR less than 3 ng/mL (Figure 2). The measured suPAR value was utilized in the treatment decision of 81.2% (178/219) of the patients.

For included patients, the median length of stay in the ED was 4.52 hours (25-75th percentile 3.48-6.14) in the intervention group and 4.9 hours ((25-75th percentile 3.19-6.38), *P* < .82) in the control group.

The median length of stay in hospital was 0 days (25-75th percentile 0-1) in the intervention group and 0 days (25-75th percentile 0-3) in the control group, (*P* = .17) (Table 2). One patient in the intervention group died of sepsis during hospital admission.

Of included patients discharged from the ED, 12,6% (23/183) returned to the ED within 7 days. Twelve ED returns were due to worsened symptoms of the same clinical issue as in the index admission and were thus considered as readmissions. Of these 12 patients, 8 patients (75%) had a suPAR value of over 3 ng/mL. The remaining 11 ED returns were not considered as readmissions: 7 ED returns were elective and 4 ED returns were due to novel clinical issues.

As mentioned earlier, under particular interest were the patient subgroups with a contradiction between the measured suPAR value and the decided follow-up treatment [(14.0% (n = 46/330)] of the whole study population), that is, discharged patients with particularly high suPAR value (>6 ng/mL) and admitted patients with particularly low suPAR value (<3 ng/mL) (Figure 2).

When observing these subgroups more closely, apart from 2 cases, the admitted patients (n = 26) with low suPAR value were all admitted for a reasonable cause such as acute appendicitis, neutropenic infection, prolonged migraine, myocardial infarction or suspected pulmonary embolism. On the contrary, a discharge should have been considered instead of an admission in the 2 exceptional cases in this subgroup (7.7% (2/26)).

On the other hand, in 90% (n = 18/20) of the 20 discharged patients with high suPAR value, an underlying chronic disease, such as chronic renal or liver insufficiency, was found to be behind the elevated suPAR value. Specific clinical reasons for the elevated suPAR values were not found in the remaining 10% (n = 2/20) of the cases.

## Discussion

The study is an interventional trial evaluating the expediency of suPAR values in the risk stratification and decision-making in the ED.

The primary outcome of the study being the number of discharges from the ED (within 24 hours of index admission), we found no significant difference between the intervention and control group. Furthermore, no statistically significant differences between the 2 groups were found in the secondary clinical outcomes such as relative proportions of hospital admissions, mortality rates and the median length of stay in the ED. A statistically significant difference between the 2 groups was found in the length of stay in the hospital. However, worth mentioning is that the utilization of suPAR did not lengthen the stays in neither the ED nor in the hospital. The study results additionally suggest that despite of not having an effect on the positive clinical outcomes, the utilization of suPAR did not increase the risk for any negative outcomes such as mortality or readmissions.

Regardless of whether suPAR was utilized or not utilized in the decision-making, patients with high (>6 ng/mL) suPAR values were more likely to be admitted to hospital (71.4% vs 29.9%) and patients with low (<3 ng/mL) suPAR values were more likely to be discharged from the ED (70.1% vs 28.6%). Additionally, 75% of the readmitted patients had a suPAR value of over 3 ng/mL. These differences between the suPAR groups indicate the biomarker’s negative and positive predicting values.

However, a significant proportion of the patients with low suPAR values were still admitted to hospital (29.9%), in other words, patient safety could be endangered if suPAR was used as a sole biomarker. Consequently, suPAR values should be used together with other clinical factors such as clinical examination findings, laboratory results and risk assessment scores. In this context, further research is needed to evaluate suPAR’s expediency.

Our study results confirmed that primary healthy patients generally had a low suPAR value. Therefore, for example, acute appendicitis did not elevate the suPAR value, which complicated the utilization of the value as a support in the decision-making in this patient group. Nevertheless, high suPAR levels have been observed to be beneficial in diagnosed appendicitis in the demonstration of the severity of the illness and in the differentiation of the cases with complications.^21^ Other urgent-care-demanding diseases not significantly raising suPAR values must be researched to more precisely evaluate the applicability of suPAR in their clinical assessment.

Our study additionally confirmed that patients with chronic diseases such as renal insufficiency had higher suPAR values. Therefore, the interpretation of suPAR values should be done by comparing the results to the patient’s former suPAR measurements.

The study results suggest that 2 admitted patients with low (<3 ng/mL) measured suPAR values were admitted with no qualified evidence. Otherwise speaking, 2 more discharges could have been made in addition to the 183 discharges during the 2-week period, thus making the total amount of discharges 185 and raising the amount by 1.1%. When observed from a larger point of view, with approximately 300 000 patients arriving at the EDs of Helsinki University Hospital yearly, a raise of this kind would allow the discharge of additional 3300 ED patients every year. However, this conclusion is subject to a high degree of uncertainty. Thus, for a reliable evaluation, a thorough analysis of the cost reductions, efficacy and safety of suPAR incorporation is needed.

Worth mentioning is that our study used an unselected population of patients arriving at the ED, reflecting that the utilization of suPAR as a prognostic biomarker did not have a significant effect in this particular population from the aspect of our study’s outcomes. Recent study suggests that in a more precisely determined patient population consisting principally of geriatric patients with nonspecific complaints (NSC), suPAR could predict 30-day mortality.^24^ That said, instead of a wide selection of patients presenting with specific symptoms such as signs of acute appendicitis, the benefits of suPAR would more likely emerge in more precisely determined target patient populations of which the risk assessment remains unclear after using the existing risk stratification methods.

### Limitations

The trial has several limitations. First, as a research environment, the ED appears quite challenging: the flow of patients is significantly high and the lengths of stay short. Moreover, in Finland, the turnover rate of the physicians of the ED is remarkably high due to the way of being on call: the EDs have an alternating population of physicians, which posed practical difficulties in the familiarization of the physicians.

Second, the study was performed during the COVID-19 pandemic, which brought up challenges in many aspects: the changing, uncertain circumstances in the ED created a novel research environment, which made the practical adjustment of the study setting more challenging and shortened the time of the study performance from 4 to 2 weeks. Moreover, due to the pandemic, recruitment of an excessive employee for research management was not possible. Finally, the logistics between the COVID-19-suspected patients and the researchers were complicated due to the prevalent isolation circumstances.

Third, the total amount of included patients was significantly lower than expected: only 30% of the evaluated study population. The small data complicated both the interpretation and evaluation of significance of the results.

Finally, as suPAR should be used together with other clinical information such as clinical examination findings, scores and laboratory results, our study only investigated suPAR independently. That said, a conclusion can be made that our study’s results appear more directional than absolute.

## Conclusion

In summary, we found that the incorporation of suPAR measurement into routine blood sampling and clinical decision-making did not have a significant effect on neither the number of discharges from the ED nor the length of stays in the ED or in the hospital. However, the utilization of suPAR did not increase the risk for any negative outcomes and did not at least lengthen the stays in the ED or in the hospital.

On a larger scale, the utilization of low suPAR values could support an additional yearly discharge of over 3300 patients in the EDs of Helsinki University Hospitals. These patients would receive more value for their visit in the ED when not admitted “just to be on the safe side..” This efficacy and the safety of suPAR incorporation must be further evaluated.

Despite of not being beneficial in our study’s unselected population, the prognostic value of suPAR could be found in the assessment of patients of whom the risk stratification remains unclear after using the existing methods through supporting the decision of either discharging or admitting the patient to hospital. We still need to determine which patient group would benefit the most.

## Supplemental Material

sj-jpg-1-bmi-10.1177_11772719221081789 – Supplemental material for Utilization of Prognostic Biomarker Soluble Urokinase Plasminogen Activator Receptor in the Emergency Department: A Tool for Safe and More Efficient Decision-making Click here for additional data file. Supplemental material, sj-jpg-1-bmi-10.1177_11772719221081789 for Utilization of Prognostic Biomarker Soluble Urokinase Plasminogen Activator Receptor in the Emergency Department: A Tool for Safe and More Efficient Decision-making by Ria M Holstein, Marja T Mäkinen, Maaret K Castrén and Johanna M Kaartinen in Biomarker Insights
