# Supplementary figures and images for: Utilization of Prognostic Biomarker Soluble Urokinase Plasminogen Activator Receptor in the Emergency Department: A Tool for Safe and More Efficient Decision-making
Source: Biomark Insights. 2022 Mar 9;17:11772719221081789. doi: 10.1177/11772719221081789 (PMC8918965; doi:10.1177/11772719221081789)

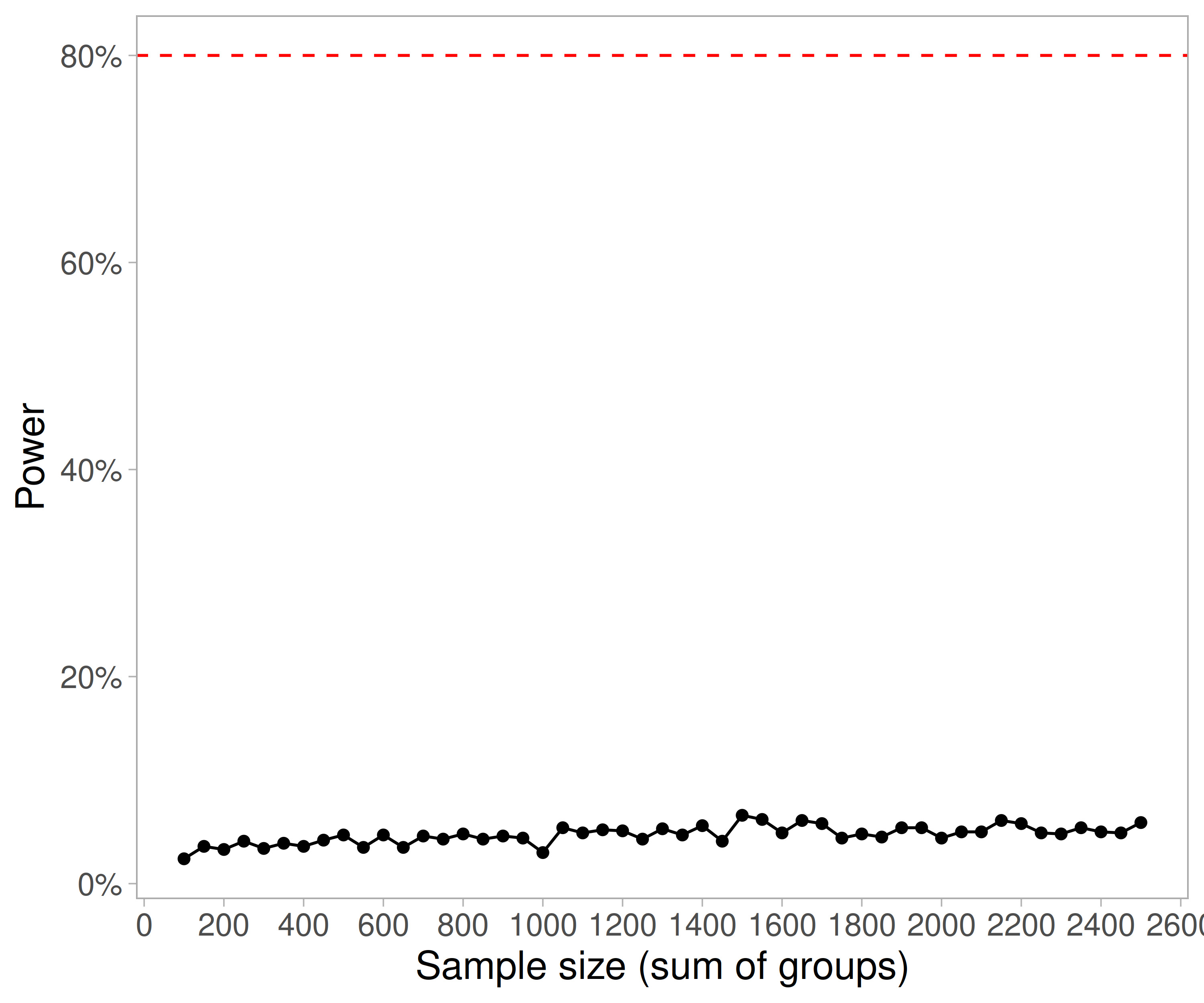

Supplement: sj-jpg-1-bmi-10.1177_11772719221081789 – Supplemental material for Utilization of Prognostic Biomarker Soluble Urokinase Plasminogen Activator Receptor in the Emergency Department: A Tool for Safe and More Efficient Decision-making [file sj-jpg-1-bmi-10.1177_11772719221081789.jpg]
